# Safety and efficacy of ripasudil in Japanese patients with glaucoma or ocular hypertension: 12-month interim analysis of ROCK-J, a post-marketing surveillance study

**DOI:** 10.1186/s12886-020-01490-1

**Published:** 2020-07-09

**Authors:** Hidenobu Tanihara, Takahiko Kakuda, Tetsuro Sano, Takashi Kanno, Ryoji Gunji

**Affiliations:** 1 Kumamoto University Hospital, Japan 1-1-1 Honjo, Chuo-ku, Kumamoto, Japan; 2 Post Marketing Surveillance Department, Kowa Co., Ltd., Tokyo, Japan

**Keywords:** Efficacy, Glaucoma, Intraocular pressure, Ripasudil, ROCK inhibitor, Safety, Observational study, Post-marketing surveillance

## Abstract

**Background:**

Ripasudil is approved in Japan for glaucoma or ocular hypertension (OH) when other treatments are ineffective or cannot be administered. Its long-term safety and efficacy are being examined in a post-marketing surveillance study; 12-month data are described here.

**Methods:**

This prospective, open-label, observational study enrolled patients with glaucoma or OH who started ripasudil during routine care. The key safety outcome was the incidence of adverse drug reactions (ADRs), focusing on allergy and/or inflammation-related ADRs such as blepharitis (including allergic) or conjunctivitis (including allergic). The primary efficacy endpoint was least squares mean (LSM) ± standard error (SE) change in intraocular pressure (IOP) from baseline to 12 months in all patients and in diagnostic groups. Secondary endpoints were change in IOP in groups stratified by treatment initiation pattern, number of concomitant drugs, and baseline IOP.

**Results:**

Overall, 3359 patients (48% male, mean age ± standard deviation [SD] 69.1 ± 12.7 years) were evaluated for safety and 3323 for efficacy. Diagnoses were primary open-angle glaucoma (43.9%), normal-tension glaucoma (36.6%), secondary glaucoma (8.7%), OH (4.2%), and primary closed-angle glaucoma (2.4%). Mean ± SD observation period was 300.1 ± 122.4 days; 1010 patients (30.1%) discontinued ripasudil by 12 months. ADRs occurred in 626 patients (18.6%); the most common were conjunctival hyperemia and blepharitis. Allergy and/or inflammation-related ADRs occurred in 388 patients (11.6%), most commonly blepharitis (5.6%) and conjunctivitis (4.2%). IOP decreased significantly from a mean ± SD 18.1 ± 6.1 mmHg at baseline; the LSM ± SE IOP change throughout 12 months of ripasudil treatment was − 2.6 ± 0.1 mmHg (− 14.0 ± 0.4%; *p* < 0.001). A significant decrease in IOP at 12 months was seen in all categories of baseline IOP (*p* < 0.001), and all types of glaucoma (*p* < 0.001), except neovascular glaucoma. Ripasudil was associated with a significant reduction in IOP at 12 months whether initiated as monotherapy or in combination with ≤4 concomitant glaucoma therapies (*p* < 0.001).

**Conclusions:**

Ripasudil was safe and effective in patients with glaucoma or OH during routine care. No new safety signals were identified, and significant reductions in IOP were maintained over 12 months.

## Background

Glaucoma is the leading cause of irreversible blindness in the world [1]. According to estimates published in 2014, between 64 and 70 million people globally are affected by glaucoma [1, 2], but as this is a condition associated with aging, the prevalence of glaucoma is estimated to increase to 76 million in 2020 and to more than 111 million by 2040 [2]. In Japan, the estimated prevalence of glaucoma is 5.0% in people ≥40 years of age [3].

Currently, the main goals of glaucoma treatment are to slow disease progression and maintain quality of life, and the only proven treatment approach is to reduce intraocular pressure (IOP) [1, 4]. International guidelines, including the Japan Glaucoma Society Guidelines for Glaucoma (fourth edition) [4], the American Academy of Ophthalmology Preferred Practice Patterns guideline [5], and guidelines from the National Institute of Health and Care Excellence in the UK [6], recommend prostaglandins as the initial medical therapy for primary open-angle glaucoma (POAG) and ocular hypertension (OH). In the Japanese guidelines, β-blockers may also be used in first-line treatment, but should be selected with due attention to contraindications and risk of adverse drug reactions (ADRs); the recommended second-line treatments are carbonic anhydrase inhibitors, α2 agonists, Rho-associated protein kinase (ROCK) inhibitors, α1 blockers, ion-channel openers, nonselective nerve stimulants, or parasympathetic nerve stimulants [4].

ROCK inhibitors reduce IOP by a different mechanism from other IOP-lowering medications; these agents increase aqueous humor outflow by reducing resistance in the trabecular meshwork [7–11]. The ROCK inhibitor ripasudil (Glanatec® ophthalmic solution 0.4%; Kowa Co. Ltd., Tokyo, Japan) was approved in Japan in September 2014 for the treatment of glaucoma and OH when other drugs are not effective or cannot be administered [11, 12]. In clinical trials, ripasudil has demonstrated IOP-lowering effects when used as monotherapy or in combination with prostaglandin analogs or β-blockers [13–17]. Ripasudil had an acceptable safety profile in these studies, with the most common ADRs being conjunctival hyperemia, blepharitis and allergic conjunctivitis [13–17]. However, there was a potential safety signal for an increased risk of allergy and/or inflammation-related ADRs with long-term treatment [15].

A long-term, large-scale post-marketing surveillance (PMS) study is being conducted in Japan to evaluate the safety and efficacy profile of ripasudil in the real-word clinical setting. Three-month interim results have been reported previously, and showed the safety and efficacy of ripasudil in reducing IOP in patients with various subtypes of glaucoma [18]. Because of the potential risk of allergy during long-term treatment with ripasudil, it is important to establish whether the short-term results seen in the 3-month analysis of the PMS study were maintained during long-term therapy. Here we report the 12-month interim results from the large-scale PMS study of ripasudil in Japan.

## Methods

### Study design and patients

This is a prospective, multicenter, open-label, PMS study investigating the safety and efficacy of ripasudil in patients with glaucoma or OH. The study design has been described in detail previously [18]. Briefly, patients with glaucoma or OH were invited to participate if they were unable to receive or responded poorly to other glaucoma medicines, had not previously received ripasudil treatment, had IOP measured before the start of ripasudil treatment, and could be registered for the study within 14 days of ripasudil initiation. Patients were instructed to instill 1 drop of ripasudil twice a day as described in the package insert, and to wait at least 5 min between instillations when using other eye drops. Eligible patients were registered for the study between June 1, 2015 and April 30, 2017 using an internet-based central registration system. The total surveillance period for this study is from June 1, 2015 to February 29, 2020, and the cut-off date for this 12-month interim analysis was September 25, 2018.

The main outcome measures are the proportion of patients with ADRs, which were classified according to the International Council for Harmonisation of Technical Requirements for Pharmaceuticals for Human Use (ICH) Medical Dictionary for Regulatory Activities (MedDRA) Version 21.0, and the mean change in IOP from baseline throughout the 12 months. An ADR was defined as an adverse event (AE) that was evaluated as treatment-related by the investigator. Key ADRs of interest were blepharitis (including allergic) and conjunctivitis (including allergic), but data on all allergic and/or inflammatory eye disorders were also collected.

IOP was assessed in the affected eye, or in the eye with the highest IOP at baseline if ripasudil was administered to both eyes. If both eyes had the same IOP at baseline, the right eye was selected for evaluation. Patients who changed glaucoma treatment after ripasudil administration, or who underwent surgery on the inner eye, were excluded from the analysis of IOP change because of the potential confounding effect of these treatments on IOP.

Patients were stratified by type of glaucoma and OH, treatment initiation patterns, baseline IOP values, and the number of active pharmaceutical ingredients used concomitantly at the start of ripasudil treatment. Patients were classified into five groups based on their previous and concomitant drug treatment: “Add-on (only)” included patients who had ripasudil alone added to their ongoing treatment with another glaucoma medicine; “Add-on (with another glaucoma drug)” included patients who had ripasudil and another glaucoma medicine simultaneously added to their ongoing treatment with another glaucoma medicine; “Switch from prior treatment” included patients who switched to ripasudil from prior treatment; “Initial monotherapy” included patients who were previously untreated and received ripasudil monotherapy as their first glaucoma treatment; and “Initial combination therapy” included patients who started their first glaucoma treatment with ripasudil in conjunction with another glaucoma medicine. The primary efficacy endpoint was mean change in IOP from baseline to 12 months in all patients and in diagnostic groups. Secondary endpoints were the mean change in IOP over 12 months in groups of patients stratified by treatment initiation pattern, number of concomitant drugs, and baseline IOP.

The study protocol was reviewed and approved by the Japanese regulatory authority prior to study initiation, and was conducted in accordance with relevant regulations in Japan (Ministerial Ordinance on Good Post-Marketing Study Practice, Ministry of Health, Labour and Welfare Ordinance Number 171, December 20, 2004). Under Japanese regulations, the protocols of PMS studies do not require review or approval by the ethics committee of the participating medical institutions or informed consent.

### Statistical analysis

The safety analysis set included all patients who returned to the clinic or hospital at least once after the start of ripasudil treatment. The efficacy analysis set included all patients from the safety analysis set who had IOP data available after the start of ripasudil treatment. Continuous efficacy variables were summarized using descriptive statistics (number of patients, mean and standard deviation [SD]), and categorical safety and efficacy variables were described using frequency and percentage. IOP values before treatment (termed baseline) were compared with IOP values obtained during the 12 months of treatment (termed post-administration). The least-squares mean (LSM) of IOP was analyzed using a mixed-effects model for repeated measures (MMRM), with IOP measurements as the objective variable and indicator function (baseline and post-administration) as the explanatory variables. The variance-covariance matrix had compound symmetry structure. The IOP changes from baseline to post-administration were shown as the LSM ± standard error (SE) with the p-value. MMRM was used to analyze change in total IOP and IOP by disease type, and also analyze change in IOP in patient subgroups based on treatment initiation pattern, IOP value before the treatment, and the number of active pharmaceutical ingredients used concomitantly. The Kaplan–Meier method was used to assess treatment continuation. All statistical analyses were performed using SAS Version 9.3 (SAS Institute, Japan).

## Results

### Patient disposition and characteristics

**Fig. 1:**
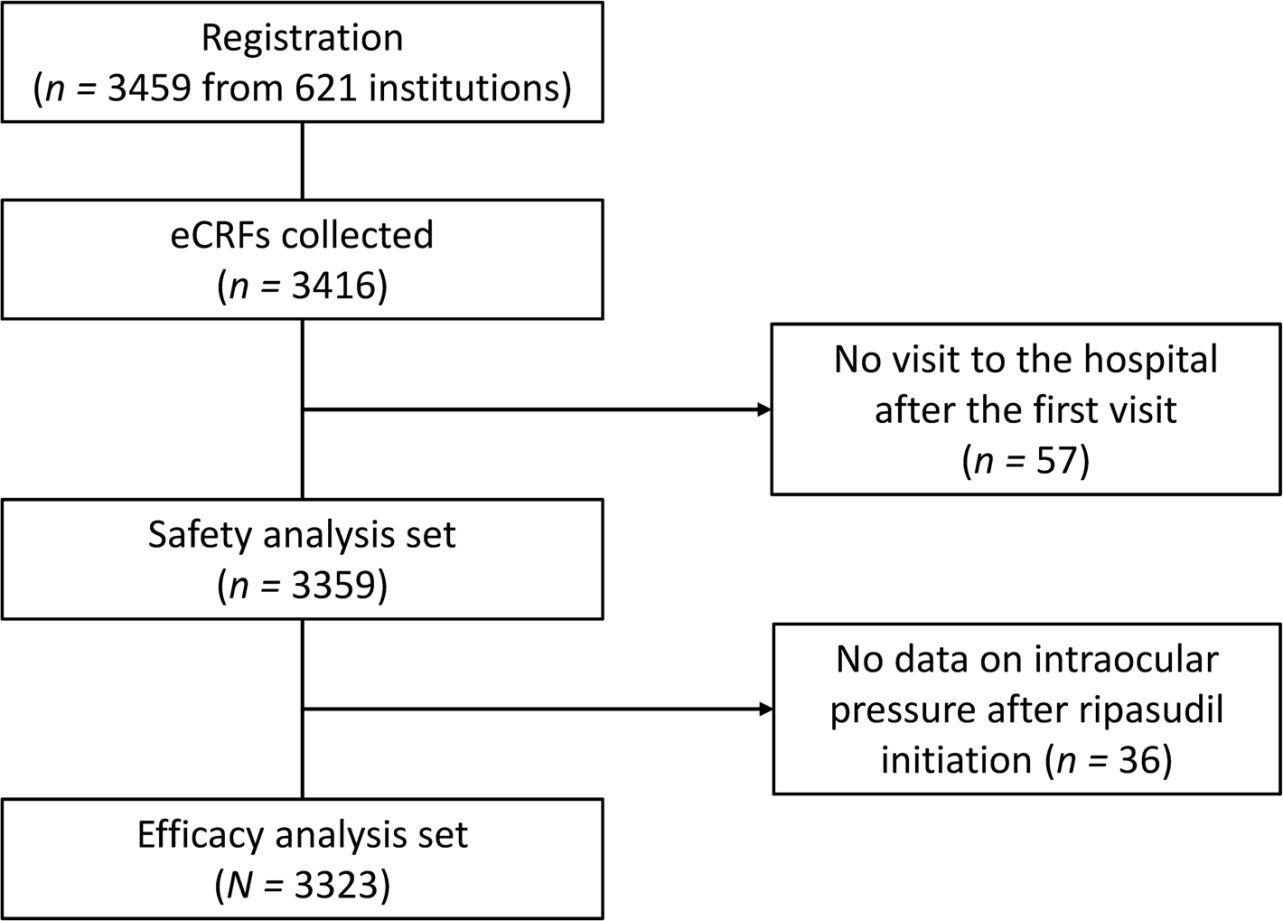
Patient flow. *eCRF* electronic case report form

Overall, 3459 patients from 621 clinical institutions were registered in the study by 30 April 2017. Of these, 3359 patients were included in the safety analysis set, and 3323 patients were included in the efficacy analysis set (Fig. 1). The mean ± SD observation period was 300.1 ± 122.4 days.

**Fig. 2:**
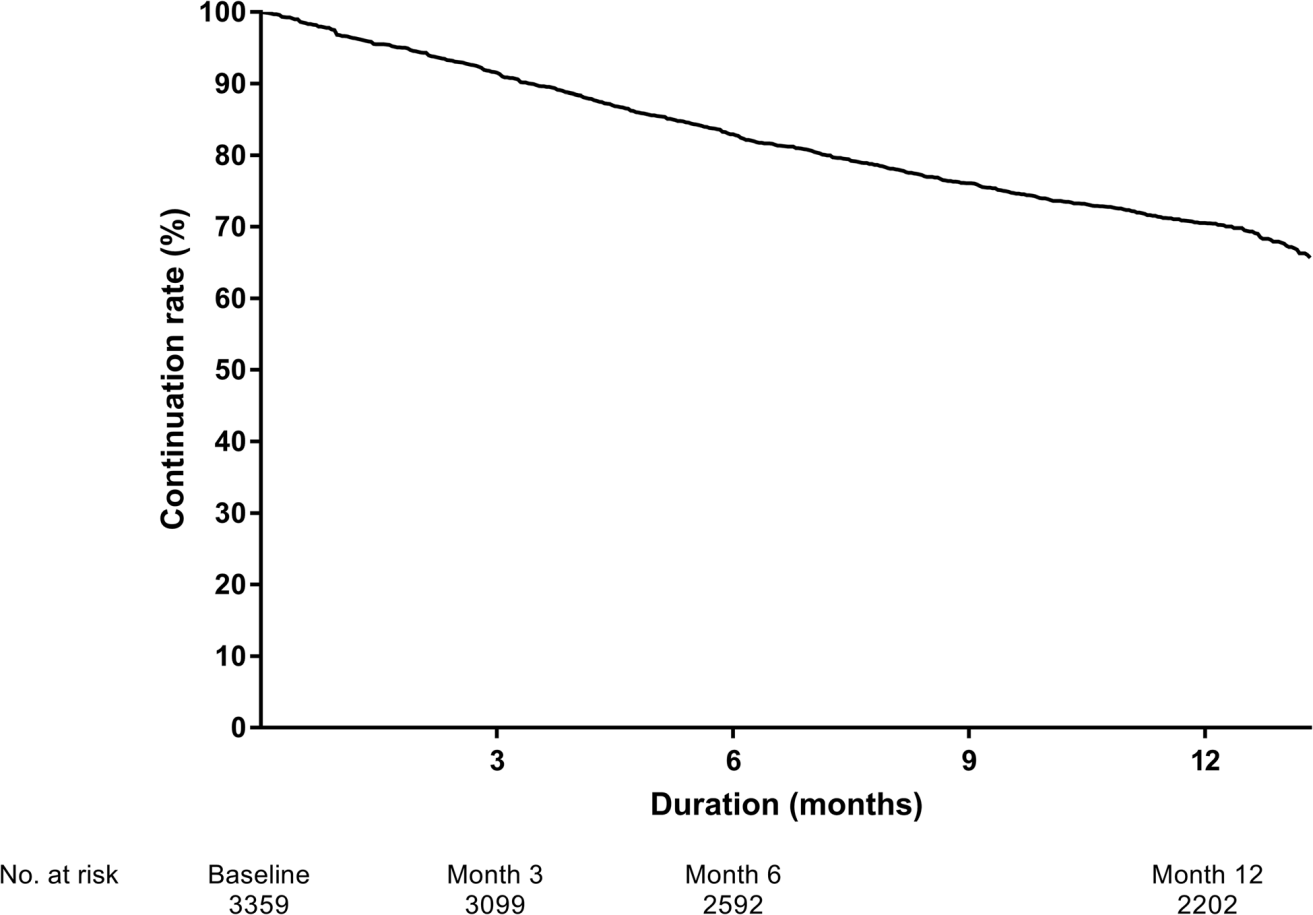
Kaplan–Meier estimate of patient discontinuation over 1 year

A total of 1010 patients (30.1%) discontinued ripasudil treatment; the main reasons for study withdrawal were AEs (*n* = 451; 13.4%), the patient stopped visiting the hospital or transferred (*n* = 246; 7.3%), and insufficient efficacy (*n* = 186; 5.5%). The continuation rate at 12 months (364 days) was 70.3% using Kaplan–Meier estimates (Fig. 2).

Approximately half of the patients in the safety analysis set were male (*n* = 1612; 48.0%), and most (70.4%) were ≥ 65 years old; mean ± SD age was 69.1 ± 12.7 years (Table 1). The most common diagnosis was POAG (43.9%), followed by normal-tension glaucoma (NTG; 36.6%) (Table 1). Secondary glaucoma was present in 8.7% of patients; the most common types were exfoliation glaucoma (3.8%), uveitis-associated glaucoma (1.7%), and steroid-induced glaucoma (1.0%).

**Table 1:** Baseline characteristics of patients

Characteristics	*N* = 3359
Sex, *n* (%)	
Male	1612 (48.0)
Female	1747 (52.0)
Age, *n* (%)	
< 65 years	994 (29.6)
≥ 65 years	2365 (70.4)
Mean ± SD, years	69.1 ± 12.7
Diagnosis, *n* (%)	
Ocular hypertension	140 (4.2)
Primary open-angle glaucoma	1475 (43.9)
Normal-tension glaucoma	1229 (36.6)
Primary angle-closure glaucoma	80 (2.4)
Secondary glaucoma	291 (8.7)
Exfoliation glaucoma	127 (3.8)
Uveitis-associated glaucoma	56 (1.7)
Steroid-induced glaucoma	32 (1.0)
Neovascular glaucoma	23 (0.7)
Childhood glaucoma	4 (0.1)
Other glaucoma	140 (4.2)

*SD* standard deviation

### Treatment initiation patterns with Ripasudil

Of the 3323 patients included in the efficacy analysis, 351 (10.6%) received ripasudil as “Initial monotherapy”, 63 (1.9%) as “Initial combination therapy”, 448 (13.5%) as “Switch from prior treatment”, 2430 (73.1%) as “Add-on (only)”, and 31 (0.9%) as “Add-on (with other glaucoma drug)”.

Most patients had been receiving prostaglandin analogs at baseline. Among patients who were taking only one active pharmaceutical ingredient for glaucoma at baseline, 85.3% were receiving prostaglandin analogs. When two active ingredients were being taken, 85.4% of patients were using a prostaglandin analog and 78.5% were using a β-blocker. In patients taking three active ingredients, these treatments were prostaglandin analogs in 96.3%, β-blockers in 93.7%, and carbonic anhydrase inhibitors in 85.9%. When four active ingredients were used, the agents were prostaglandin analogs in 99.1%, β-blockers in 99.1%, and carbonic anhydrase inhibitors in 98.8%, and α2 stimulants in 92.4%. In the group receiving five active ingredients for glaucoma, 100% were using prostaglandin analogs, 100% β-blockers, 100% carbonic anhydrase inhibitors, 80.0% α2 stimulants, 60.0% systemic carbonic anhydrase inhibitor, and 33.3% parasympathetic nerve stimulants.

### Safety

Overall, AEs were reported in 872 patients (26.0%), and ADRs in 626 patients (18.6%) (Table 2). Of the ADRs classified as eye disorders, the most common were conjunctival hyperemia (*n* = 223; 6.6%), blepharitis (*n* = 189; 5.6%), conjunctivitis (*n* = 141; 4.2%), and eye pruritus (*n* = 44; 1.3%) (Table 2). Allergy and/or inflammation-related ADRs were reported in 388 patients (11.6%) (Table 3). The most common sites for allergy- or inflammation-related ADRs were the eyelids and conjunctiva.

**Table 2:** Adverse drug reactions

Adverse drug reactions, *n* (%)	*N* = 3359
No. of patients with ADR	626 (18.6)
Eye disorders	
Conjunctival hyperemia^a^	223 (6.6)
Blepharitis^b^	189 (5.6)
Conjunctivitis^c^	141 (4.2)
Eye pruritus	44 (1.3)
Punctate keratitis	25 (0.7)
Eye pain	16 (0.5)
Vision blurred	12 (0.4)
Corneal erosion	9 (0.3)
Eye irritation	9 (0.3)
Eyelid edema	9 (0.3)
Dry eye	8 (0.2)
Erythema of eyelid	4 (0.1)
Eye discharge	4 (0.1)
Corneal disorder	4 (0.1)
Conjunctival follicles	3 (0.1)
Lacrimation increased	3 (0.1)
Eyelids pruritus	3 (0.1)
Keratitis^d^	3 (0.1)
Abnormal sensation in eye	2 (0.1)
Eyelid ptosis	2 (0.1)
Foreign body sensation in eyes	2 (0.1)
Cataract^e^	2 (0.1)
Other^f^ (incidence of each)	1 (< 0.1)
Other ADRs	
Dermatitis contact	6 (0.2)
Headache	5 (0.2)
Dermatitis allergic	3 (0.1)
Dizziness	3 (0.1)
Intraocular pressure increased	3 (0.1)
Somnolence	2 (0.1)
Nausea	2 (0.1)
Other^g^ (incidence of each)	1 (< 0.1)

*ADR* adverse drug reaction

^a^ Including ocular hyperemia

^b^ Including blepharitis allergic

^c^ Including conjunctivitis allergic

^d^ Including allergic keratitis

^e^ Including nuclear cataract

^f^ One patient each developed asthenopia, conjunctival erosion, conjunctival hemorrhage, conjunctival edema, corneal epithelium defect, corneal edema, eczema of the eyelids, macular edema, meibomianitis, scintillating scotoma, eyelid skin dryness, administration site dermatitis, hordeolum

^g^ One patient each developed erythema, pruritus, urticaria, cerebral infarction, cough, dyspnea, epistaxis, malaise, dermatitis, bradycardia, palpitations, rhinitis, otitis externa, dysgeusia

**Table 3:** Allergy and/or inflammation-related adverse drug reactions during ripasudil treatment

Allergy and/or inflammation-related ADRs, *n* (%)	*N* = 3359
No. of pts. with allergy and/or inflammation-related ADR	388 (11.6)
Blepharitis^a^	189 (5.6)
Conjunctivitis^b^	141 (4.2)
Eye pruritus	44 (1.3)
Punctate keratitis	25 (0.7)
Eyelid edema	9 (0.3)
Corneal erosion	9 (0.3)
Dermatitis contact	6 (0.2)
Erythema of eyelid	4 (0.1)
Corneal disorder	4 (0.1)
Dermatitis allergic	3 (0.1)
Conjunctival follicles	3 (0.1)
Eyelids pruritus	3 (0.1)
Keratitis	2 (0.1)
Other^c^ (incidence of each)	1 (< 0.1)

*ADR* adverse drug reaction, *pts* patients

^a^ Including blepharitis allergic

^b^ Including conjunctivitis allergic

^c^ One patient each developed conjunctival erosion, conjunctival edema, corneal epithelium defect, eczema eyelids, allergic keratitis

### Efficacy

**Fig. 3:**
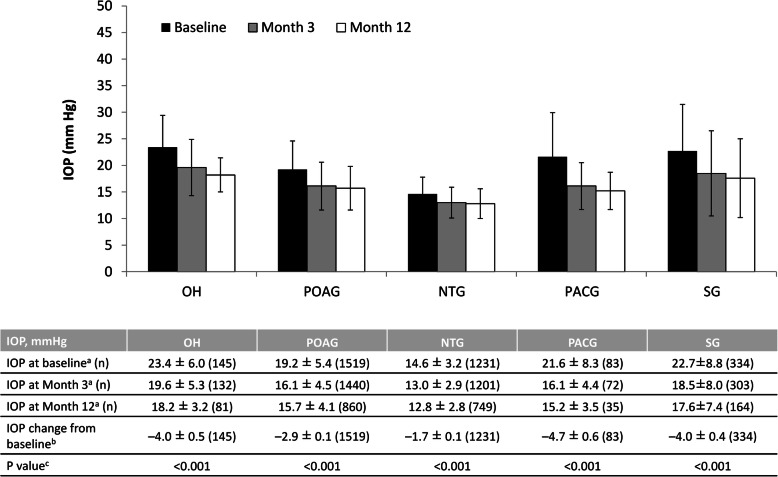
Intraocular pressure and change in intraocular pressure in patients according to glaucoma type or ocular hypertension. *IOP* intraocular pressure, *NTG* normal-tension glaucoma, *OH* ocular hypertension, *PACG* primary angle-closure glaucoma, *POAG* primary open-angle glaucoma, *SG* secondary glaucoma. ^a^ Mean ± standard deviation. ^b^ Least-squares mean ± standard error. ^c^ Mixed effects model for repeated measures, baseline vs entire post-administration period

**Fig. 4:**
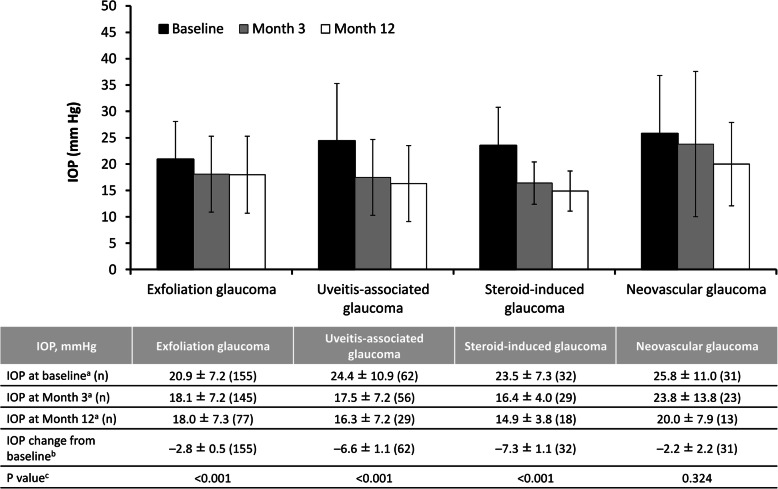
Intraocular pressure and change in intraocular pressure in patients according to the type of secondary glaucoma. *IOP* intraocular pressure. ^a^ Mean ± standard deviation. ^b^ Least-squares mean ± standard error. ^c^ Mixed effects model for repeated measures, baseline vs entire post-administration period

**Fig. 5:**
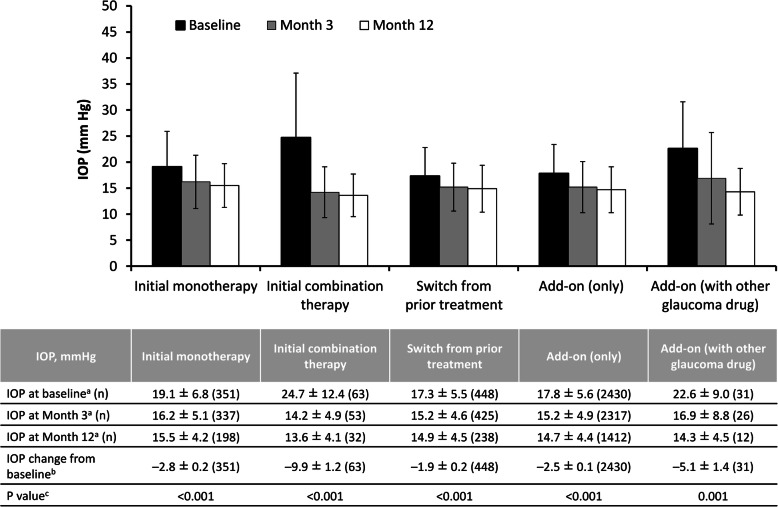
Intraocular pressure and change in intraocular pressure in patients according to treatment initiation patterns. *IOP* intraocular pressure. ^a^ Mean ± standard deviation. ^b^ Least-squares mean ± standard error. ^c^ Mixed effects model for repeated measures, baseline vs entire post-administration period

In the overall efficacy analysis set (*n* = 3323), IOP decreased from a mean ± SD of 18.1 ± 6.1 (*n* = 3323) at baseline to 15.3 ± 4.9 at month 3 (*n* = 3158), and 14.8 ± 4.4 at month 12 (*n* = 1892); the LSM ± SE change from baseline in IOP over the entire 12-month treatment period with ripasudil was − 2.6 ± 0.1 mmHg (*p* < 0.001). There was a significant decrease in IOP between baseline and 12 months in all types of glaucoma or ocular hypertension (*p* < 0.001) (Fig. 3). Among patients with secondary glaucoma, 12 months of treatment with ripasudil were associated with a significant decrease in IOP in those with exfoliation glaucoma (LSM ± SE change of − 2.8 ± 0.5 mmHg; *p* < 0.001), uveitis-associated glaucoma (LSM ± SE change of − 6.6 ± 1.1 mmHg; *p* < 0.001), and steroid-induced glaucoma (LSM ± SE change of − 7.3 ± 1.1 mmHg; *p* < 0.001), but not in those with neovascular glaucoma (LSM ± SE change of − 2.2 ± 2.2 mmHg; *p* = 0.324) (Fig. 4). Initiating treatment with ripasudil was associated with a significant reduction in IOP at 12 months regardless of the treatment initiation pattern (*p* < 0.001) (Fig. 5).

**Fig. 6:**
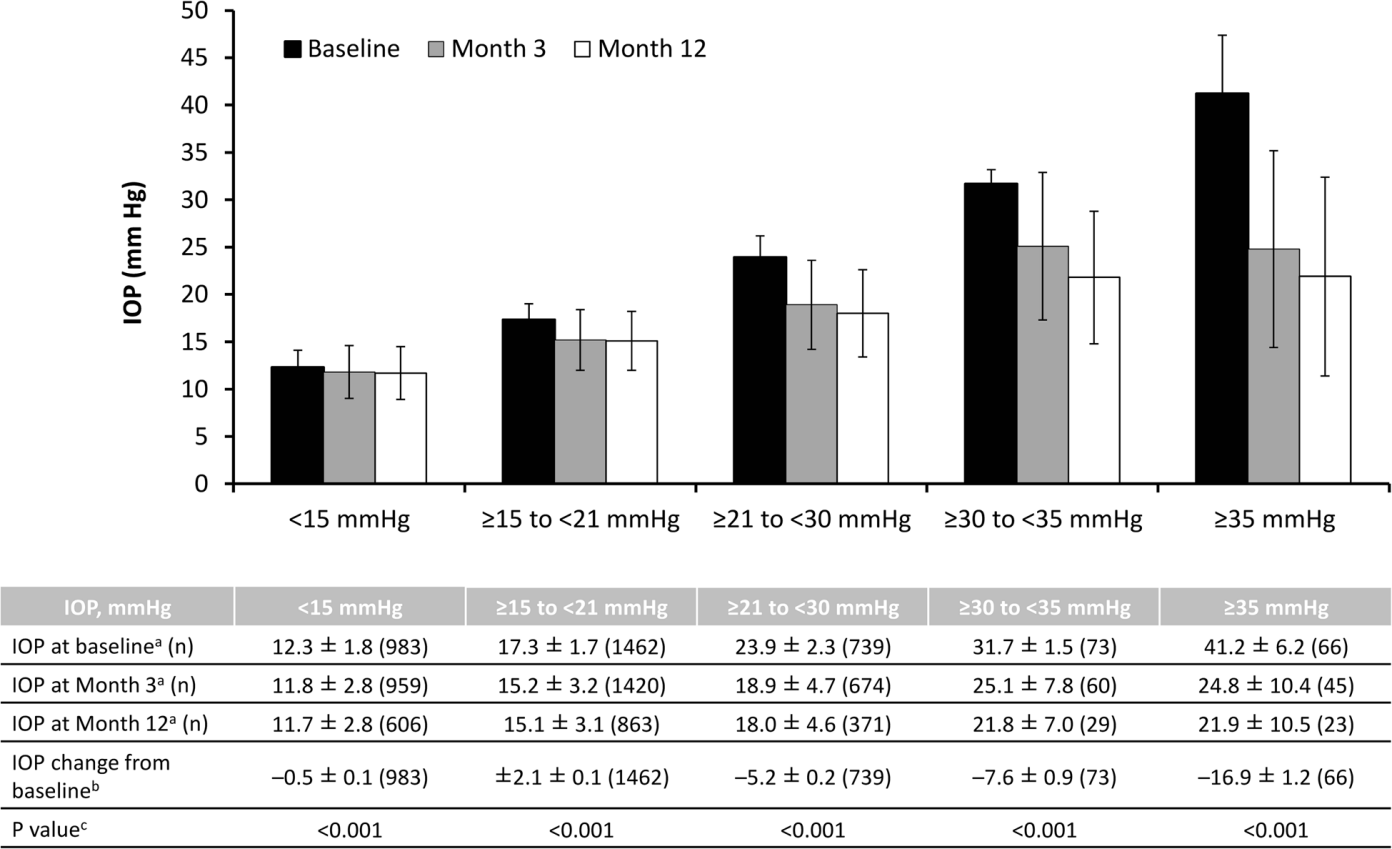
Intraocular pressure and change in intraocular pressure in all patients according to baseline intraocular pressure values. *IOP* intraocular pressure. ^a^ Mean ± standard deviation. ^b^ Least-squares mean ± standard error. ^c^ Mixed effects model for repeated measures, baseline vs entire post-administration period

The change in IOP at 3 and 12 months, stratified by baseline IOP, is shown in Fig. 6. Although all groups showed a significant reduction after 12 months of treatment with ripasudil (*p* < 0.001), the magnitude of the change was greater with increasing baseline IOP values, ranging from a LS mean ± SE change of − 0.5 ± 0.1 mmHg in patients with a baseline IOP of < 15 mmHg to a LS mean ± SE change of − 16.9 ± 1.2 mmHg in those with a baseline IOP of ≥35 mmHg.

**Fig. 7:**
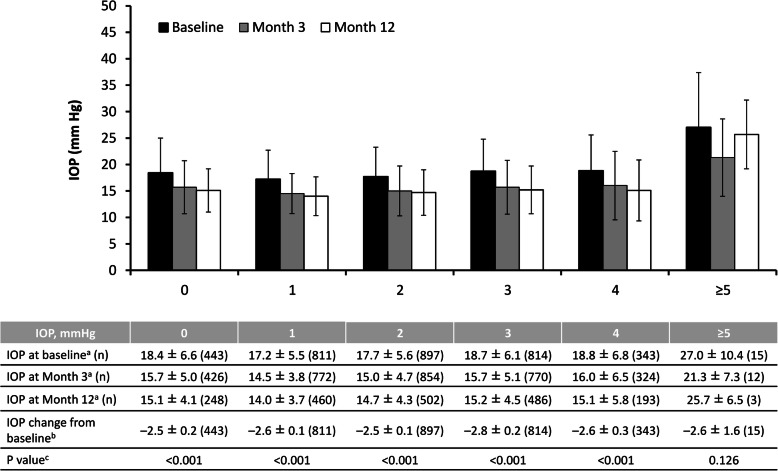
Intraocular pressure and change in intraocular pressure in all patients according to the number of active pharmaceutical ingredients concomitantly used. *IOP* intraocular pressure. ^a^ Mean ± standard deviation. ^b^ Least-squares mean ± standard error. ^c^ Mixed effects model for repeated measures, baseline vs entire post-administration period

Figure 7 shows the change from baseline in IOP stratified by the number of concomitant active pharmaceutical ingredients for glaucoma that the patients were receiving. The change was significant (*p* < 0.001) throughout 12 months in all groups except for those who were receiving ≥5 concomitant active ingredients, although it should be noted that only three patients in this group had 12-month IOP data.

## Discussion

The current 12-month interim analysis provides long-term data on the safety and efficacy of ripasudil in a large group of patients (> 3000) receiving treatment in a real-world clinical practice setting. Our results confirm the earlier results from the 3-month interim analysis of this PMS study [18], showing that ripasudil is effective in lowering IOP across a range of patients, and that the lack of safety issues seen during short-term treatment is also true during treatment for 12 months. While no major safety issues were found in this study with ripasudil for 12 months, ongoing data collection is important, as is continued monitoring of potential safety issues in patients on ripasudil.

In this PMS study, approximately 30.1% of patients withdrew from the study – a reasonable proportion considering the real-world nature of this study. This percentage is lower than in a previous Japanese study, which reported that 39.1% of patients who start topical therapy with antiglaucoma medication discontinue such treatment within 12 months [19]. The most common reasons for discontinuation in our study were development of an AE (13.4%), the patient stopped visiting the hospital or transferred (7.3%), and insufficient efficacy (5.5%). It should be noted that discontinuation due to AEs is not only based on the judgment of the physician but also on the judgment of the patient; therefore, it may include discontinuation due to cataract surgery (which is not necessarily causally related to ripasudil). The reasons why patients stopped visiting the hospital or transferred are not known, as this information is not recorded during routine clinical practice. For patients with glaucoma, lack of knowledge and/or skepticism can negatively impact adherence, which is an important risk factor for progression of the disease [20].

The incidence of ADRs over 12 months of treatment was 18.6% and the incidence of allergy- and/or inflammation-related ADRs was 11.6%. The most common ADRs were blepharitis (including allergic blepharitis), which developed in 5.6% of patients, and conjunctivitis (including allergic forms), which developed in 4.2%. The incidence of ADRs in the current PMS study was low compared with data from our previous long-term uncontrolled clinical trials with ripasudil, which reported blepharitis in 17.8% of patients and allergic conjunctivitis in 15.3% [15].

In the phase III long-term study, there was no consistent trend in the time from the start of ripasudil administration to the onset of blepharitis and allergic conjunctivitis, and there was no relationship between the time to onset of these ADRs and their severity [15]. Similarly, in the current study, no consistent trend was found in the timing of onset of allergy- and/or inflammation-related ADRs during long-term administration.

There may be a number of reasons for the low incidence of ADRs seen in the current study compared with previous research. First, controlled clinical trials that proactively follow up patients on a regular protocol-defined schedule tend to identify more ADRs than studies with less active surveillance (such as our study in patients who were seen in routine clinical practice) [21]. Second, clinically minor AEs may be overlooked by patients. Research suggests that only about 50% of patients who develop an AE in routine clinical practice report the event to their physician, and only ~ 20% of AEs are documented by physicians in the medical records [22]. Third, patients were informed before the study that ripasudil can cause conjunctival hyperemia, so they may not have reported this event as an ADR. Furthermore, it is possible that the conjunctival hyperemia was transient and disappeared in the time between application and examination [23]. Therefore, while the incidence of ADRs may have been underestimated in this study, the reported incidence likely reflects those ADRs that are bothersome enough to patients that they prompt a report to their physicians. In this regard, fewer patients than we had anticipated reported bothersome ADRs or discontinued treatment because of AEs.

Corneal disorders were rare in the current study. The recent phase III ROCKET-1 and -2 studies with the ROCK inhibitor netarsudil reported corneal verticillata in 8.8% of patients receiving netarsudil alone and in 14.6% of patients receiving netarsudil in combination with timolol [24]. Moreover, in ROCKET-2, approximately 25% of patients who used netarsudil once or twice daily for 12 months developed corneal verticillata [25]. In animal models and in vitro studies, ripasudil induced transient morphological changes in corneal endothelial cells, but these changes favored corneal wound healing [26]. Consistent with this, none of the patients receiving ripasudil in the current study developed corneal verticillata, and there were no ADRs suggestive of corneal endothelial dysfunction (e.g. corneal opacification, corneal edema or bullous keratopathy) in patients who received ripasudil for 12 months.

This study also found that ripasudil significantly reduced IOP, with the change apparent at the 3-month assessment and maintained over 12 months of treatment. The significant reduction in IOP was seen in almost all subgroups when patients were stratified by treatment initiation pattern, glaucoma subtype, baseline IOP and number of concomitant active ingredients. Subgroups in which no significant change in IOP between baseline and 12 months was detected (i.e. those with neovascular glaucoma and those receiving ≥5 concomitant medications at baseline) contained fewer than 15 patients, and were therefore probably underpowered to detect a significant treatment difference. As noted in the 3-month study results, there was an absence of significant IOP reduction in patients with neovascular glaucoma, an effect thought to be a result of the pathogenesis of the disease [18]. This phenomenon may be explained by the fact that, in the pathogenesis of neovascular glaucoma, the trabecular meshwork is seriously damaged by the proliferation of neovascular tissue [27]. It is interesting to note that adding ripasudil to treatment with up to four other glaucoma active ingredients still resulted in a significant decrease in IOP between baseline and 12 months. The most common concomitant medications were prostaglandin analogs, β-blockers, carbonic anhydrase inhibitors and α2 stimulants. These data suggest that a ROCK inhibitor may provide a valuable add-on treatment option to any glaucoma medication combination because it has a distinct mechanism of action.

This study has a number of limitations. First, as an observational study, there is a potential for selection bias (and/or regression to the mean) and there was no control group comparator. Similarly, as an observational study, all treatment decisions, including when and how to initiate treatment, choice of combination therapies, discontinuation or switching, were at the treating physician’s discretion, which complicates analysis of efficacy and safety. Second, as the study is ongoing, it is possible that the findings may differ at the 24-month assessment as a result of further data retrieval and analysis.

## Conclusions

The current study demonstrates the safety and efficacy of ripasudil over 12 months in patients with glaucoma or ocular hypertension receiving this agent during routine clinical care. No new safety signals or major safety concerns were identified, and significant reductions in IOP that were apparent at 3 months were maintained over 12 months of treatment. This 12-month interim analysis of a PMS study suggests that ripasudil is a promising agent for the treatment of glaucoma when used in various dosage forms or in combination with other glaucoma therapies.

## Data Availability

The datasets generated during and/or analyzed during the current study are available from the corresponding author on reasonable request.

## Abbreviations

ADR: Adverse drug reaction
AE: Adverse event
eCRF: Electronic case report form
ICH: International Council for Harmonisation of Technical Requirements for Pharmaceuticals for Human Use
IOP: Intraocular pressure
LSM: Least squares mean
MedDRA: Medical Dictionary for Regulatory Activities
MMRM: Mixed-effects model for repeated measures
NTG: Normal-tension glaucoma
OH: Ocular hypertension
PACG: Primary angle-closure glaucoma
PMS: Post-marketing surveillance
POAG: Primary open-angle glaucoma
ROCK: Rho-associated protein kinase
SD: Standard deviation
SE: standard error
SG: Secondary glaucoma
