# Correction to: Safety and efficacy of ripasudil in Japanese patients with glaucoma or ocular hypertension: 12-month interim analysis of ROCK-J, a post-marketing surveillance study

**DOI:** 10.1186/s12886-020-01595-7

**Published:** 2020-09-04

**Authors:** Hidenobu Tanihara, Takahiko Kakuda, Tetsuro Sano, Takashi Kanno, Ryoji Gunji

**Affiliations:** 1 Kumamoto University Hospital, Japan 1-1-1 Honjo, Chuo-ku, Kumamoto, Japan; 2 Post Marketing Surveillance Department, Kowa Co., Ltd., Tokyo, Japan

**Correction to: BMC Ophthalmol 20, 275 (2020)**

**https://doi.org/10.1186/s12886-020-01490-1**

Following publication of the original article [1] the authors have notified us of an error in the Fig. 6. The corrected Fig. 6 is presented below:

**Fig. 6:**
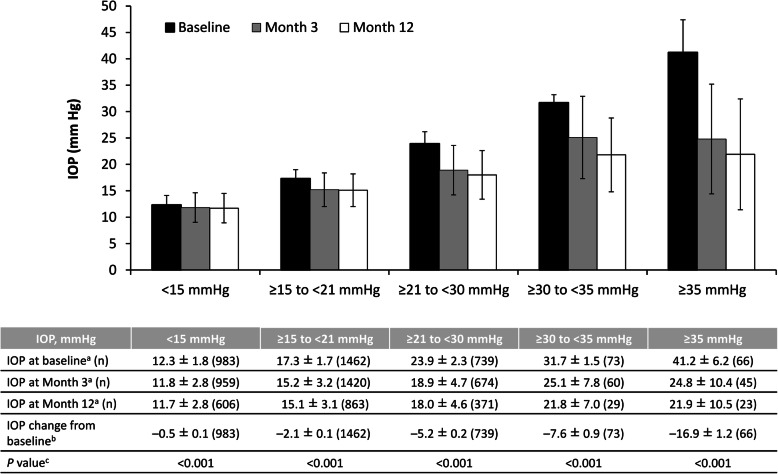
Intraocular pressure and change in intraocular pressure in all patients according to baseline intraocular pressure values. *IOP* intraocular pressure. ^a^ Mean ± standard deviation. ^b^ Least-squares mean ± standard error. ^c^ Mixed effects model for repeated measures, baseline vs entire post-administration period
